# Supplementary material for: A benchmark of computational pipelines for single-cell histone modification data
Source: Genome Biol. 2023 Jun 20;24:143. doi: 10.1186/s13059-023-02981-2 (PMC10280832; doi:10.1186/s13059-023-02981-2)
Supplement: Supplementary file 1 — Additional file 1. Contains the supplementary figures, tables, and text. [file 13059_2023_2981_MOESM1_ESM.pdf]

Additional file 1 for: A benchmark of computational pipelines for single-cell histone modification data

Félix Raimundo[1, 3], Pacôme Prompsy [2, 3], Jean-Philippe Vert[1,*,†] and Céline Vallot[2,3,†]

[1] Google Research, Brain team, 75009 Paris, France
[2] CNRS UMR3244, Institut Curie, PSL Research University, 75005 Paris, France
[3] Translational Research Department, Institut Curie, PSL Research University, 75005 Paris, France

May 11, 2023

*Now at Owkin, 75010, Paris, France
†Correspondance: celine.vallot@curie.fr and jean-philippe.vert@owkin.com

Supplementary material

Supplementary text

Role of whitening in LSI

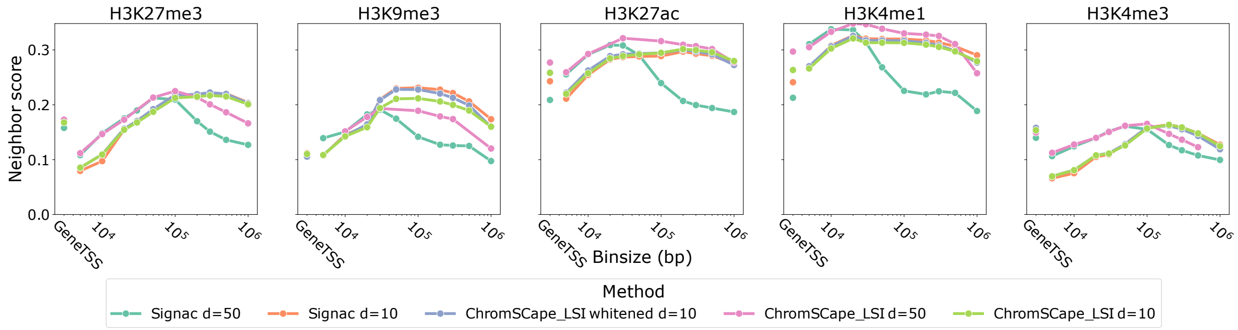

Figure S1: Performances of Signac, ChromSCape_LSI, and modified ChromSCape_LSI on the 5 marks in the mouse brain dataset, as a function of the matrix construction

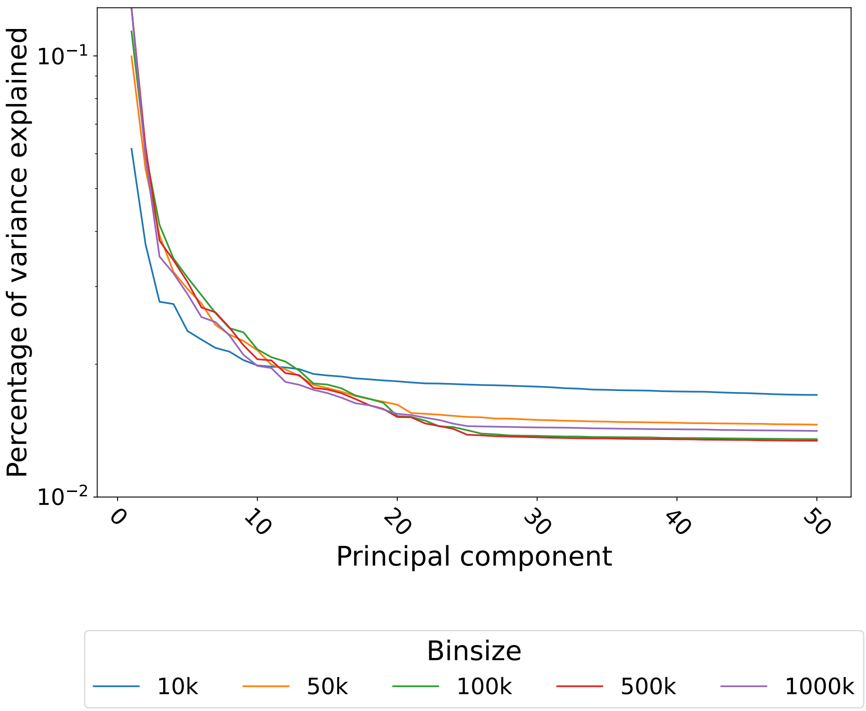

Figure S2: Percentage of variance explained by each principal component of LSI on H3K4me1 of the mouse brain dataset.

We could see in Fig. 3 that Signac's best performances are achieved for a smaller bin size than the other methods, it is especially surprising because it is supposed to implement the same algorithm as ChromSCape_LSI. In this section we investigate the reason behind this difference.

A close look at the implementation of Signac shows that it does not implement the standard LSI algorithm, but instead a whitened version of it; the principal components (PC) are not weighted by their explained variance. Furthermore the default dimension used is 50 instead of 10 for ChromSCape_LSI. Both methods also remove the first

PC, as it is assumed to be mostly driven by the coverage per cell. In order to understand the cause of the shift in optimal bin size we ran Signac with dimension 10, and ran ChromSCape_LSI at dimension 50 and modified it to use whitening.

By comparing the three conditions with a dimension of 10, we can see that the difference in performances between Signac and ChromSCape_LSI in the previous sections can mostly be explained by the number of PCs, indeed except in the case of H3K9me3 the performances of the two are almost identical as can be seen in Fig. S1.

However we can see that in higher dimensions, 50 PCs, the performances are very different. While the top performances are comparable, with a slight advantage for ChromSCape_LSI, Signac has a much tighter range of bin sizes with good performances. This can be explained by the fact that in large dimensions, the later PCs explain less variance than the early ones, and not weighing appropriately induces noise. We can see that when the bin size is small, the explained variance per PC is less concentrated in the first PCs than in the later ones, this is shown in Fig. S2. This explains why Signac has a tighter range of good bin sizes, as its whitening makes the later PCs as important as the first ones.

We thus recommend not whitening when using LSI, as it makes the method less robust to the choice of bin size.

Trade-off between coverage and cell number

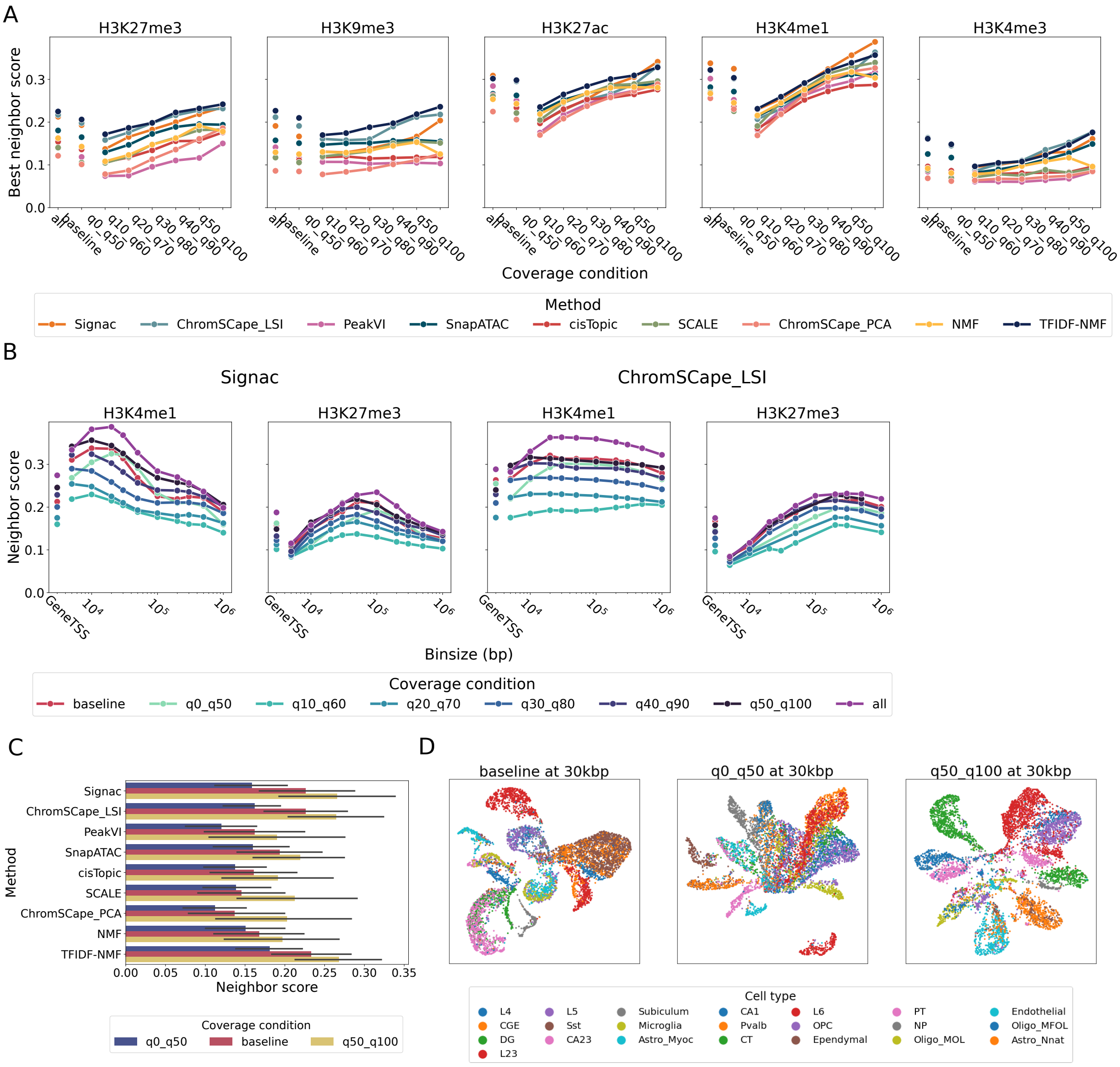

Figure S3: Study of the effect of cell coverage on the performances of the representations. The all condition contains all the cell as a reference, the baseline condition contains only 50% of the cells uniformly sampled at random, the other 6 conditions contain 50% of the cells, but are sorted by coverage. We order the cells by how much reads they contain and take all the cells from the bottom n% up to $n + 50\%$ in order to have the same amount of cells in all conditions. **A** Best performance across matrix construction, measured for all of the 9 methods, 5 marks of the mouse brain dataset and 8 coverage conditions. **B**. Performances of Signac and ChromSCape_LSI on H3K4me1 and H3K27me3 as a function of matrix construction. **C**. Average best performance of the 9 methods across all marks, for the lowest covered cells, random cells, and highest covered cells. **D.** UMAP projection of H3K4me1 at different coverage qualities, using ChromSCape_LSI across at 30kbp, colored by the labels of [1] obtained from the scRNA-seq co-assays.

While experimentalists do not control the depth of sequencing per cell with the current technologies, we felt that studying how increasing the coverage would impact the quality of the representations to be of interest. Indeed we currently do not know whether there would be a benefit from such an increase, and if there is one, how strong its effect

would be.

In order to evaluate the effect of coverage, we select 50% of the cells, but constrain them to have similar coverage. For example in the q0_q50 condition we take the 50% cells with the lowest coverage per cell, and in q50_q100 we take the cells with the highest coverage. In a more general way, we sort the cells by coverage per cell, and select the cells whose coverage falls between the n-th percentile, and $n + 50$-th percentile. This allows us to have all conditions with the same amount of cells, and just study the effect of coverage. We also have a condition where we just sample half of the cells at random, including all coverage, in order to have a baseline to compare against. That protocol is summarized in Fig. S3. This approach of sampling the cells by coverage instead of downsampling the reads per cell has the advantage that it does not make any assumption on the data generation process. Indeed here all the observed cells are real cells, instead of cells that are modified with computational means.

First we can observe that the performances of all methods increase as we increase the coverage, which was expected. However unlike the number of cells in an experiment seen in Fig. 6, the positive effect of more reads per cells does not plateau and is almost a straight line in the case of H3K4me1 as we can see in Fig. S3. If we look at the differences in performances between the least and most covered cells, summarized by mark in Table S12 and by method in Table S13, we can see an increase of at least 35%. That increase even goes as far as 107% for H3K4me3 with ChromSCape_LSI. Looking at the difference between the baseline and the high coverage cells, that increase is still in the order of 15%. It is interesting to notice that this effect on performances is larger than the one obtained by an increase in the number of cells, furthermore as we can see in Fig. S3 this gain is not yet completely achieved in our protocol. That effect is specifically noticeable for H3K27me3. This also agrees with the results on the section studying the role of selecting cells by coverage, where we identified that the marks benefiting the most from this selection were H3K27me3, H3K27ac, and H3K4me1.

The effect of coverage is also consistent across methods, with Signac, ChromSCape_LSI an ChromSCape_PCA benefiting the most form this increase in coverage, above 60%. The first two already being the best performing methods allows to fully reap the benefits from a high coverage.

Supplementary tables

	H3K27me3	H3K9me3	H3K27ac	H3K4me1	H3K4me3
Signac	88.2%	88.5%	90.3%	93.6%	84.8%
ChromSCape_LSI	80.0%	80.3%	78.8%	93.9%	78.2%
PeakVI	86.1%	87.9%	85.2%	84.5%	77.3%
SnapATAC	88.2%	89.1%	90.6%	94.8%	84.8%
cisTopic	88.2%	88.5%	90.3%	93.6%	84.8%
SCALE	86.1%	87.3%	90.6%	92.1%	85.2%
ChromSCape_PCA	88.2%	88.5%	90.3%	93.6%	84.8%
NMF	100%	100%	100%	100%	100%
TFIDF-NMF	100%	100%	100%	100%	100%

Table S1: Percentage of successful runs on the mouse brain data.

	H3K27me3	H3K9me3	H3K27ac	H3K4me1	H3K4me3
Signac	91.7%	91.7%	91.7%	91.7%	83.3%
ChromSCape_LSI	83.3%	83.3%	83.3%	83.3%	83.3%
PeakVI	83.3%	50.0%	75.0%	83.3%	91.7%
SnapATAC	91.7%	91.7%	8.3%	58.3%	91.7%
cisTopic	75.0%	75.0%	75.0%	75.0%	75.0%
SCALE	75.0%	50.0%	83.3%	83.3%	83.3%
ChromSCape_PCA	83.3%	83.3%	83.3%	83.3%	83.3%
NMF	100%	100%	100%	100%	100%
TFIDF-NMF	100%	100%	100%	100%	100%

Table S2: Percentage of successful runs on the human PBMC data.

Method	neighbor score				
	H3K27me3	H3K9me3	H3K27ac	H3K4me1	H3K4me3
Signac	0.213	0.191	**0.309**	**0.338**	0.161
ChromSCape_LSI	0.217	0.212	0.302	0.321	**0.164**
PeakVI	0.159	0.141	0.284	0.302	0.084
SnapATAC	0.180	0.157	0.266	0.282	0.125
cisTopic	0.154	0.130	0.263	0.267	0.096
SCALE	0.140	0.117	0.261	0.270	0.088
ChromSCape_PCA	0.121	0.086	0.225	0.256	0.069
NMF	0.163	0.129	0.254	0.267	0.092
TFIDF-NMF	**0.225**	**0.227**	0.302	0.322	0.161

Table S3: Best performance of each method across feature engineering methods on the mouse brain dataset, the best performing method for each mark is bolded.

Method	ratio
Chromscape_LSI	1.886
Chromscape_PCA	1.855
PeakVI	1.793
SCALE	1.449
Signac	1.796
SnapATAC	1.445
cisTopic	1.399
NMF	1.757
TFIDF-NMF	1.265

Table S4: Ratio between the best and worst performances for each method across matrix construction, with no preprocessing, averaged over the mouse brain dataset marks.

Mark	ratio
H3K27ac	1.479
H3K27me3	1.987
H3K4me1	1.487
H3K4me3	1.586
H3K9me3	1.596

Table S5: Ratio between the best and worst performances for each mark of the mouse brain dataset across matrix construction, with no preprocessing, averaged over the 9 methods.

	H3K27me3	H3K9me3	H3K27ac	H3K4me1	H3K4me3
Signac	1.96	1.96	1.65	1.79	1.62
ChromSCape_LSI	2.54	1.95	1.37	1.22	2.35
PeakVI	2.83	1.57	1.65	1.36	1.57
SnapATAC	1.58	1.61	1.28	1.37	1.38
cisTopic	1.43	1.35	1.35	1.54	1.33
SCALE	1.80	1.33	1.38	1.43	1.30
ChromSCape_PCA	2.01	1.49	1.94	2.14	1.70
NMF	2.55	1.67	1.60	1.45	1.52
TFIDF-NMF	1.18	1.45	1.10	1.09	1.52

Table S6: Ratio between the best and worst performances across matrix construction on the raw data (no feature or cell selection applied) for each mark and method combination on the mouse brain dataset.

	H3K27me3	H3K9me3	H3K27ac	H3K4me1	H3K4me3
Signac	2.78	5.16	4.41	2.47	1.18
ChromSCape_LSI	1.30	1.56	1.52	1.25	1.46
PeakVI	1.20	3.95	5.07	7.64	1.10
SnapATAC	2.65	2.36	1.00	3.19	2.57
cisTopic	1.04	1.12	1.18	1.19	1.40
SCALE	2.00	2.14	2.22	3.19	1.12
ChromSCape_PCA	1.14	1.32	1.48	1.27	1.07
NMF	5.60	5.87	5.79	8.99	5.39
TFIDF-NMF	5.16	6.03	7.00	10.76	6.08

Table S7: Ratio between the best and worst performances across matrix construction on the raw data (no feature or cell selection applied) for each mark and method combination on the human PBMC dataset.

Mark	best increase Signac	best increase ChromSCape_LSI
H3K27me3	1.104	1.071
H3K9me3	1.073	1.043
H3K27ac	1.110	1.095
H3K4me1	1.149	1.133
H3K4me3	1.008	1.082

Table S8: Ratio of the performances between the best coverage threshold and the worst one for each mark on the mouse brain dataset.

Method	best increase
Chromscape_LSI	1.085
Chromscape_PCA	1.409
PeakVI	1.076
SCALE	1.211
Signac	1.089
SnapATAC	1.098
cisTopic	1.082
TFIDF-NMF	1.092
cisTopic	1.072

Table S9: Ratio of the performances between between the best coverage threshold and the worst one, averaged by method on the mouse brain dataset.

	Signac		ChromSCape_LSI	
Mark	From 40%	From 60%	From 40%	From 60%
H3K27ac	1.091	1.047	1.088	1.028
H3K27me3	1.155	1.061	1.118	1.084
H3K9me3	1.143	1.089	1.096	1.017
H3K4me1	1.057	1.013	1.057	1.025
H3K4me3	1.172	1.081	1.185	1.075

Table S10: Ratio of the performances, averaged by mark, between having all the cells present and having either only 40% or 60% of themin the mouse brain dataset.

Method	From 40%	From 60%
Chromscape_LSI	1.109	1.046
Chromscape_PCA	1.188	1.092
PeakVI	1.342	1.180
SCALE	1.290	1.159
Signac	1.124	1.058
SnapATAC	1.084	1.038
cisTopic	1.210	1.108

Table S11: Ratio of the performances, averaged by method, between having all the cells present and having either only 40% or 60% of them in the mouse brain dataset.

	Signac		ChromSCape_LSI	
Mark	large increase	baseline	large increase	baseline
H3K27ac	1.708	1.150	1.666	1.119
H3K27me3	1.711	1.216	1.464	1.170
H3K9me3	1.555	1.223	1.355	1.140
H3K4me1	1.688	1.193	1.754	1.204
H3K4me3	1.682	1.087	2.067	1.220

Table S12: Ratio of the performances between having high coverage (q50_100 condition) in the mouse brain dataset, and either low coverage or baseline coverage, averaged by mark. The "large increase" column is the increase in performance observed against q0_50 where we select the cells with the lowest coverage. The "baseline" column is the increase against no selection.

Method	large increase	baseline
Chromscape_LSI	1.661	1.170
Chromscape_PCA	1.791	1.502
PeakVI	1.561	1.152
SCALE	1.504	1.467
Signac	1.669	1.174
SnapATAC	1.421	1.143
cisTopic	1.361	1.174

Table S13: Ratio of the performances between having high coverage (q50_100 condition) in the mouse brain dataset, and either low coverage or baseline coverage, averaged by method. The "large increase" column is the increase in performance observed against q0_50 where we select the cells with the lowest coverage. The "baseline" column is the increase against no selection.

Supplementary Figures

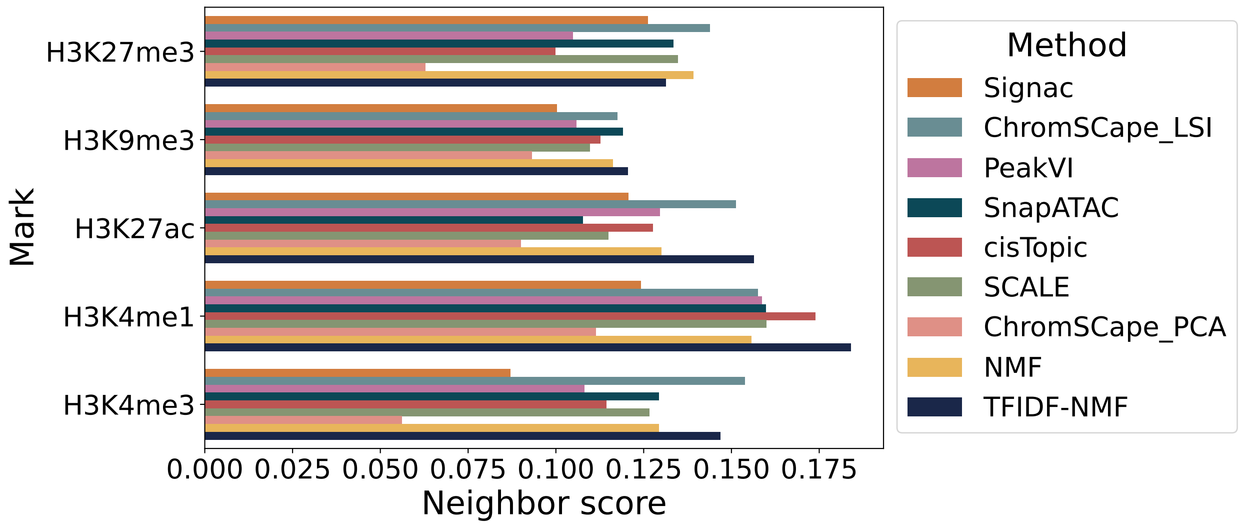

Figure S4: Best performances of the different representation methods on the human PBMC dataset.

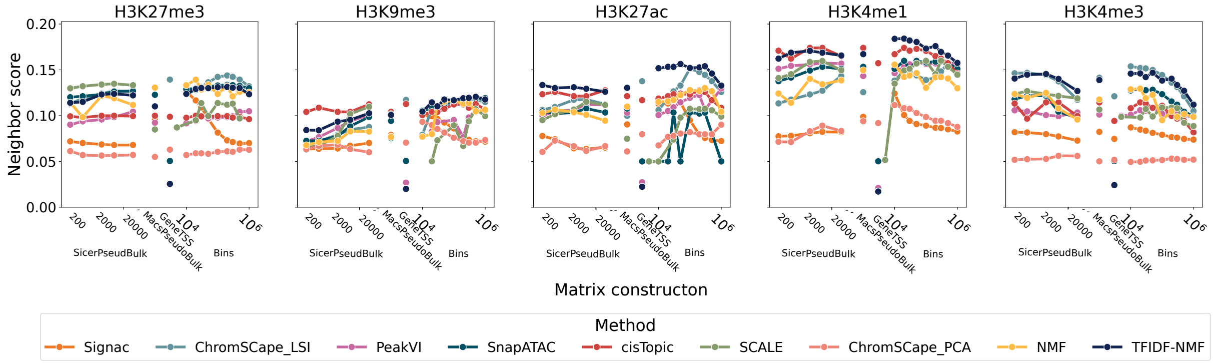

Figure S5: Neighbor score of the 9 dimension reduction algorithms on the 5 marks in the human PBMC dataset, as a function of the matrix construction. The values in SicerPseudoBulk correspond to different width parameters for the islands, the values in Bins correspond to the various binsizes used.

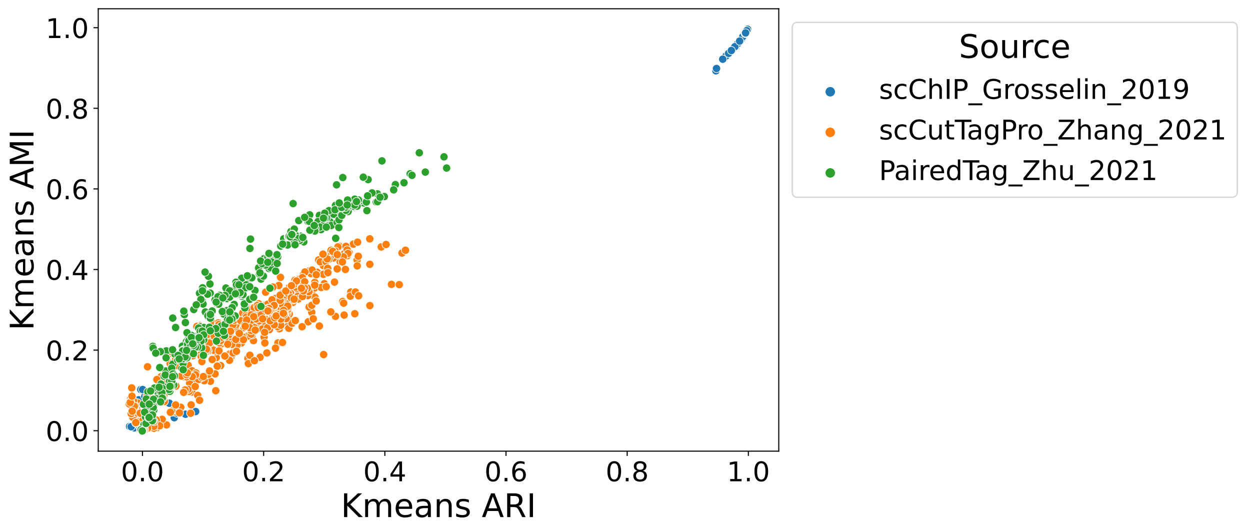

Figure S6: Correlation of ARI using k-means on the different datasets.

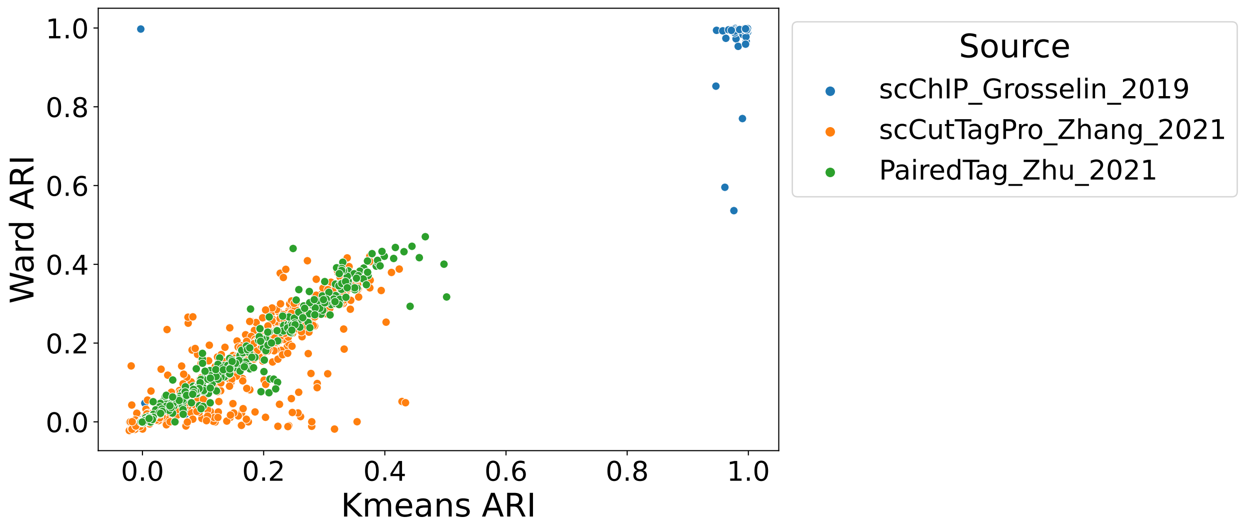

Figure S7: Correlation of ARI using k-means or Ward on the different datasets.

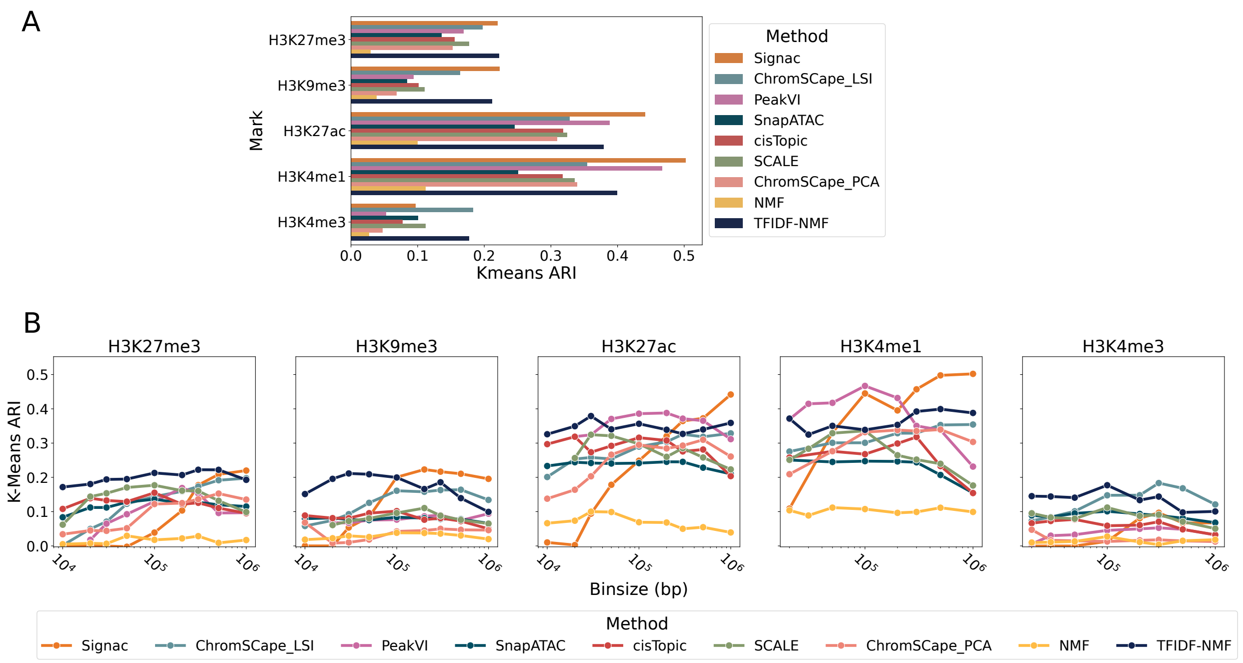

Figure S8: ARI of the 9 dimension reduction algorithms on the 5 marks in the mouse brain dataset. **A** Best performances across the various matrix construction methods. **B** Performances as a function of the matrix construction.

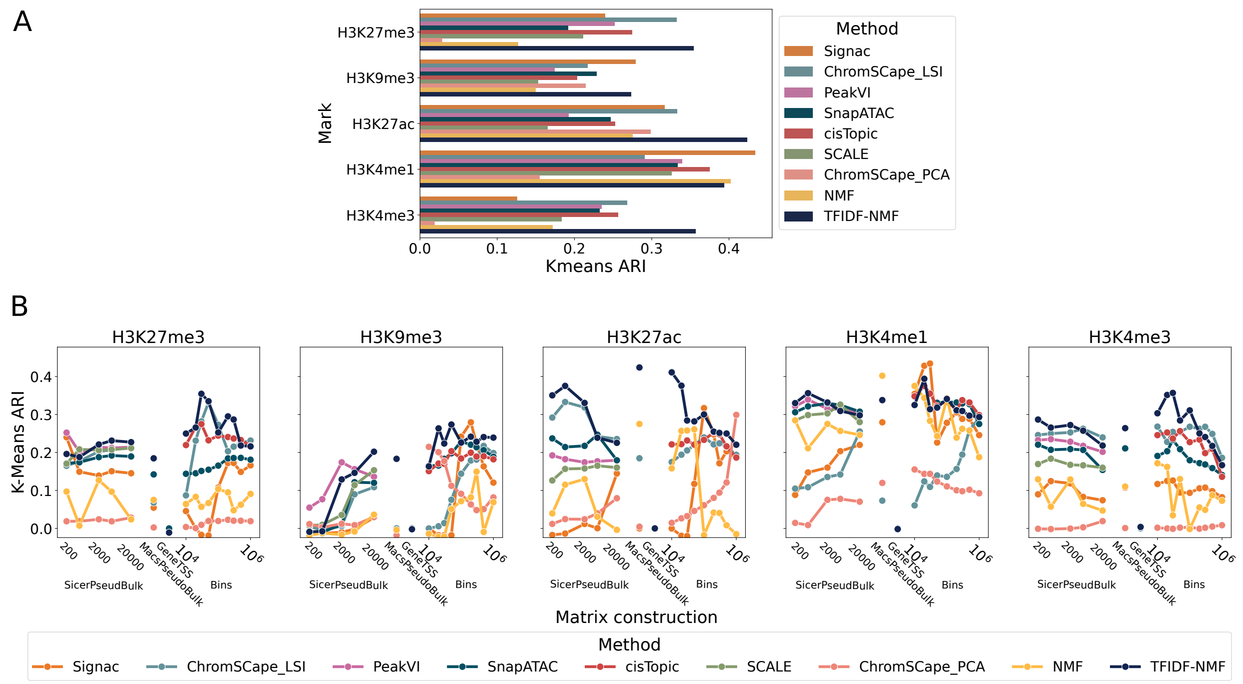

Figure S9: ARI of the 9 dimension reduction algorithms on the 5 marks in the human PBMC dataset. **A** Best performances across the various matrix construction methods. **B** Performances as a function of the matrix construction.

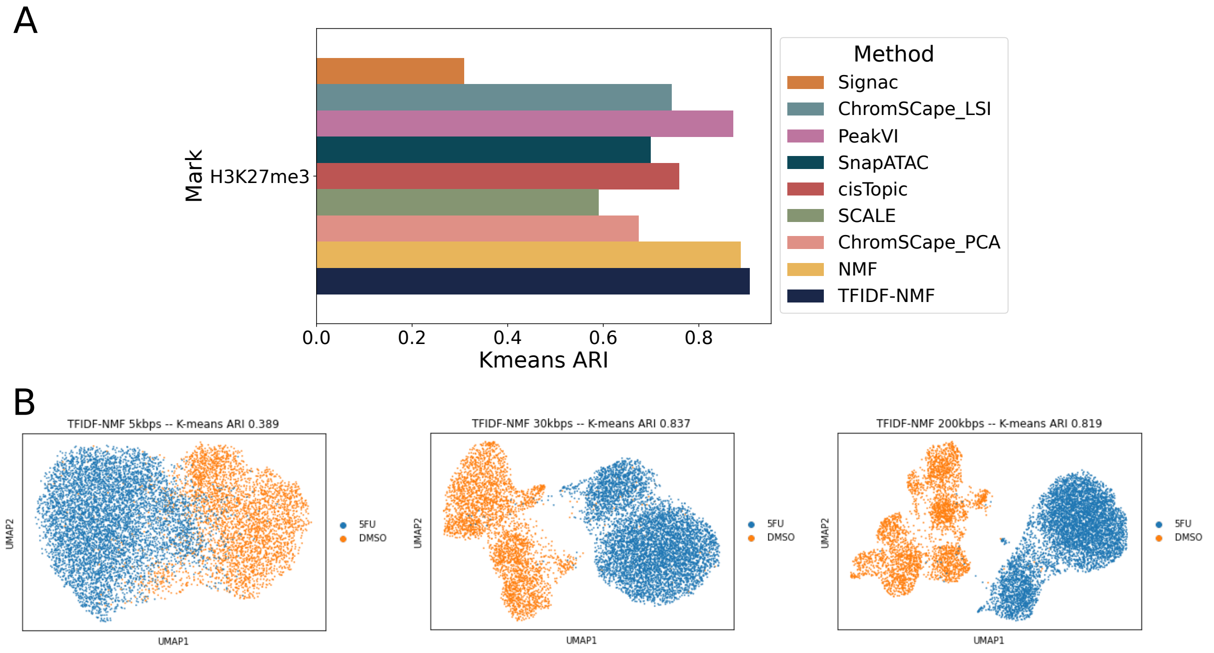

Figure S10: **A.** Best performances achieved by each method across all matrix constructions. **B.** UMAP projection at various binsizes for TFIDF-NMF coloured by treatment status.

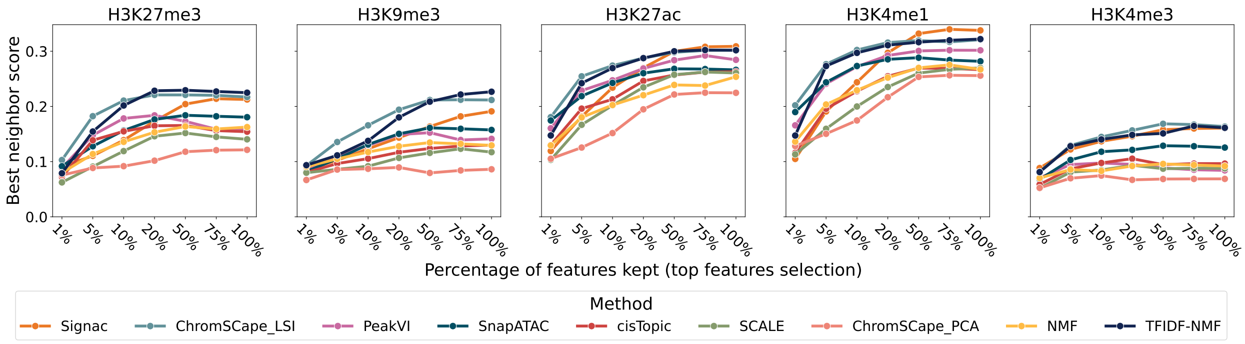

Figure S11: Role of feature selection, using the top features method used for scRNA-seq Each point corresponds to the best performance across matrix construction of a given method and a given percentage of features kept, for the 9 methods, 5 marks, and 7 features selection conditions

References

[1] Zhu, C., Zhang, Y., Li, Y., Lucero, J., Behrens, M. & Ren, B. Joint profiling of histone modifications and transcriptome in single cells from mouse brain. *Nature Methods*. **18**, 283-292 (2021)
